# Practical quantification of image registration accuracy following the AAPM TG‐132 report framework

**DOI:** 10.1002/acm2.12348

**Published:** 2018-06-07

**Authors:** Kujtim Latifi, Jimmy Caudell, Geoffrey Zhang, Dylan Hunt, Eduardo G. Moros, Vladimir Feygelman

**Affiliations:** ^1^ Department of Radiation Oncology Moffitt Cancer Center Tampa FL USA

**Keywords:** deformable image registration, rigid image registration, validation of image registration

## Abstract

The AAPM TG 132 Report enumerates important steps for validation of the medical image registration process. While the Report outlines the general goals and criteria for the tests, specific implementation may be obscure to the wider clinical audience. We endeavored to provide a detailed step‐by‐step description of the quantitative tests’ execution, applied as an example to a commercial software package (Mirada Medical, Oxford, UK), while striving for simplicity and utilization of readily available software. We demonstrated how the rigid registration data could be easily extracted from the DICOM registration object and used, following some simple matrix math, to quantify accuracy of rigid translations and rotations. The options for validating deformable image registration (DIR) were enumerated, and it was shown that the most practically viable ones are comparison of propagated internal landmark points on the published datasets, or of segmented contours that can be generated locally. The multimodal rigid registration in our example did not always result in the desired registration error below ½ voxel size, but was considered acceptable with the maximum errors under 1.3 mm and 1°. The DIR target registration errors in the thorax based on internal landmarks were far in excess of the Report recommendations of 2 mm average and 5 mm maximum. On the other hand, evaluation of the DIR major organs’ contours propagation demonstrated good agreement for lung and abdomen (Dice Similarity Coefficients, DSC, averaged over all cases and structures of 0.92 ± 0.05 and 0.91 ± 0.06, respectively), and fair agreement for Head and Neck (average DSC = 0.73 ± 0.14). The average for head and neck is reduced by small volume structures such as pharyngeal constrictor muscles. Even these relatively simple tests show that commercial registration algorithms cannot be automatically assumed sufficiently accurate for all applications. Formalized task‐specific accuracy quantification should be expected from the vendors.

## 1 INTRODUCTION

Image registration is currently widely used in radiation oncology clinical practice. However, it is a complex subject, and image registration software, such as treatment planning and other radiotherapy software, has to undergo acceptance testing and validation to assess its performance and limitations prior to clinical use. The AAPM TG 132 Report on “Use of image registration and fusion algorithms and techniques in radiotherapy”^1^ (the Report) enumerates important steps for validation and verification of the image registration process. Furthermore, the supplemental materials in the Report contain a series of publicly available image datasets designed to help in quantitating image registration accuracy. While the Report outlines the general goals and criteria for the tests, specific implementation may be obscure to the wider clinical audience. Certain tests are not accompanied by readily available software to implement them. In this paper, we endeavored to provide a detailed step‐by‐step description of the quantitative tests’ (Section 4.C of the Report) execution, striving for simplicity and utilization of software either in the public domain, or ubiquitous in general (e.g., Microsoft Excel) or in radiotherapy (e.g., a treatment planning system). We illustrate our approach by applying the tests suggested in the Report to a commercial image registration software package that may have been less explored in the radiotherapy literature in comparison with others.

## 2 METHODS

### 2.A Image registration software

As an example of an image registration software package, we used Mirada RTx v. 1.6 (Mirada Medical, Oxford, UK), which is currently in clinical service at our institution. It has a rigid registration algorithm and two choices for deformable image registration (DIR). The rigid registration is based on the Mutual Information^2^, ^3^, ^4^, ^5^ approach and has a number of spatial resolution settings. The finest grid was always used. The DIR portion includes two algorithms. One (“CT Deformable”) is used for CT to CT registration when the datasets are similar, and is a derivative of Lucas‐Kanade optical flow algorithm.^6^ For CT datasets with dissimilar intensities and cross‐modality registration, the “Multimodality Deformable” option is used, which optimizes a Mutual Information‐based similarity function.^3^, ^7^, ^8^ The software is capable of exporting Digital Imaging and Communications in Medicine (DICOM) spatial registration objects for both rigid and deformable registrations. The deformation vector field (DVF) is downsampled spatially compared to the imaging datasets themselves, by a factor of 2 in each dimension for “CT Deformable” and a factor of 4 for “Multimodality Deformable”.

### 2.B Quantification of registration errors

#### 2.B.1 DICOM transformation objects

**Figure 1:**
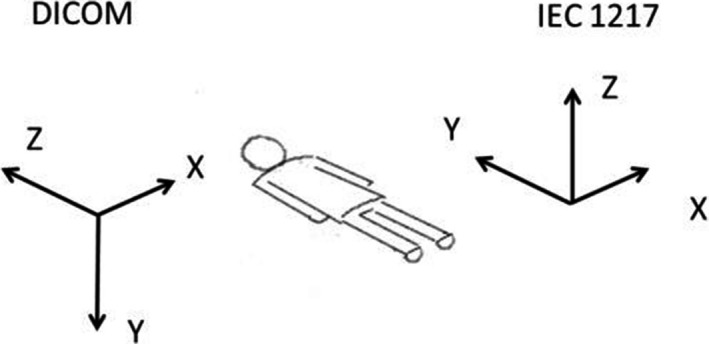
Relationship between the patient‐based DICOM and room‐based IEC1217 coordinate systems for a patient in a standard position (HFS).

Before describing the methods of quantifying registration errors, it is instructive to reiterate some pertinent details of the DICOM standard.^9^ The DICOM spatial frame of reference convention differs from the one typically employed in the modern treatment planning systems and linear accelerators (e.g., IEC1217). It is a right‐handed patient‐based coordinate system. The relationship between the DICOM and IEC1217 systems for a patient in a standard (head first supine, or HFS) position is depicted in Fig. 1. The DICOM coordinate system is employed exclusively throughout this paper.

A 4 × 4 homogeneous transformation matrix that registers a coordinate system A to B has the following form:^9^

(1) $$\left[\begin{array}{c} A_{x}\\ A_{y}\\ A_{z}\\ 1 \end{array}\right]=\left[\begin{array}{cccc} R_{11} & R_{12} & R_{13} & T_{x}\\ R_{21} & R_{22} & R_{23} & T_{y}\\ R_{31} & R_{32} & R_{33} & T_{z}\\ 0 & 0 & 0 & 1 \end{array}\right]\left[\begin{array}{c} B_{x}\\ B_{y}\\ B_{z}\\ 1 \end{array}\right]$$

Vectors ***A*** and ***B*** are the coordinates of a point in two respective reference frames, vector ***T*** represents translations, and matrix ***R*** is the 3‐dimensional rotational transformation matrix. The last row of ones and zeroes has no physical meaning but is rather added for consistency of matrix operations. This matrix can be easily extracted from the DICOM rigid registration object with any text editor. Although the file is binary, the matrix values are visible as a string of 16 slash‐separated ASCII — represented numbers ending in “0/0/0/1”. The 4 × 4 matrix from eq. (1) is streamed row‐by‐row (row‐major). For the translation only cases, the rotational matrix is an identity one, and only the translational vector is meaningful.

The deformable registration DICOM object, in its essence, contains the DVF, called Vector Grid Data. It is a binary stream of data encoding the magnitude and direction of displacement of the center of each voxel $\left(\Delta x_{\mathrm{ijk}},\Delta y_{\mathrm{ijk}},\Delta z_{\mathrm{ijk}}\right)$. The displacement operation can be preceded and/or followed by optional pre‐ and postdisplacement rigid transformations described by eq. (1).

#### 2.B.2 Rigid translations

Quantification of the translational‐only registration errors is straightforward. The known values of ***T*** in eq. (1), based on the applied shifts described in the Report for Basic Phantom and Basic Anatomical Datasets are presented in Table 1. These nominal ***T*** values within each dataset group do not change, since the different modality images share the reference frame with the phantom CT. The signs of the expected ***T*** values take into account the fact that eq. (1) reports a transformation from the target to the moving dataset, which is opposite to the registration direction. The difference between the nominal ***T*** values from Table 1 and those reported by the registration software are the errors along the cardinal axes that can be compared to the corresponding image voxel sizes.

**Table 1:** Rigid registration tests — translations only. The data are combined from Tables 5 and 6 in the Report

Case	Stationary dataset	Moving dataset	Known shifts	Known ***T** (x,y,z)* (mm)
1	Basic Phantom Dataset 2 (CT)	Basic Phantom Dataset 1 (CT)	Dataset 2 is shifted wrt Dataset 1 by 10 mm to patient Lt, 5 mm Ant, 15 mm Sup.	(−10, 5, −15)
2		Basic Phantom Dataset 1 (PET)		
3		Basic Phantom Dataset 1 (MR1)		
4		Basic Phantom Dataset 1 (MR2)		
5		Basic Phantom Dataset 1 (CBCT)		
6	Basic Anatomical Dataset 1 (CT)	Basic Anatomical Dataset 2 (CT)	Datasets 2,3,4,5,6 shifted wrt Dataset 1 by 3 mm Lt, 5 mm Ant, 12 mm Sup.	(3, −5, 12)
7		Basic Anatomical Dataset 3 (PET)		
8		Basic Anatomical Dataset 4 (MRT1)		
9		Basic Anatomical Dataset 5 (MRT2)		

#### 2.B.3 Rigid translations and rotations

Rigid registration involving translations and rotations is slightly more complicated. The tests are enumerated in Table 2, along with the known translations. Note that the known ***T*** values in Table 2 differ not only in sign but also in magnitude from the nominal X,Y,Z shifts specified in the Report. The reason for that is that in the transformation calculations, rotations are applied first, followed by translations. To determine the known ***T*** values, we first independently construct a direct transformation matrix ***M*** from the moving to stationary datasets, corresponding to the known rotations and shifts in Table 2. While the order of the rotations is not explicit in the report, it was determined by trial and error to be around the Z axis first, followed by Y, and finally X. In matrix notation, this implies:

(2) $$R=R_{x}R_{y}R_{z}$$

**Table 2:** Rigid registration tests — translations and rotations

Case	Stationary dataset	Moving dataset	Known shifts	Known rotations	Known ***T** (x,y,z)* (mm)
10	Basic Phantom Dataset 3 (CT)	Basic Phantom Dataset 1 (CT)	Dataset 3 is shifted wrt Dataset 1 by 5 mm to patient Lt, 15 mm Ant, 20 mm Sup.	−5°around *X*‐axis, 8° Y, 10° Z	(−5.07, 17.29, −18.06)
11		Basic Phantom Dataset 1 (PET)			
12		Basic Phantom Dataset 1 (MR1)			
13		Basic Phantom Dataset 1 (MR2)			
14		Basic Phantom Dataset 1 (CBCT)			

For the transformation in Cases 10–14, the individual rotational components are

(3) $$\begin{array}{cc} & R_{x}=\left[\begin{array}{ccc} 1 & 0 & 0\\ 0 & \text{cos}(-5^{\circ }) & -\text{sin}(-5^{\circ })\\ 0 & \text{sin}(-5^{\circ }) & \text{cos}(-5^{\circ }) \end{array}\right]\\ & R_{y}=\left[\begin{array}{ccc} \text{cos}(8^{\circ }) & 0 & -\text{sin}(8^{\circ })\\ 0 & 1 & 0\\ \text{sin}(8^{\circ }) & 0 & \text{cos}(8^{\circ }) \end{array}\right]\\ & R_{z}=\left[\begin{array}{ccc} \text{cos}(10^{\circ }) & -\text{sin}(10^{\circ }) & 0\\ \text{sin}(10^{\circ }) & \text{cos}(10^{\circ }) & 0\\ 0 & 0 & 1 \end{array}\right] \end{array}$$

After matrix multiplication and insertion of the translational vector, the numerical transformation matrix becomes:

(4) $$\begin{array}{c} M=\left[\begin{array}{cccc} 0.975 & -0.162 & -0.152 & 5\\ 0.172 & 0.983 & 0.062 & -15\\ 0.340 & -0.086 & 0.986 & 20\\ 0 & 0 & 0 & 1 \end{array}\right] \end{array}$$

To compare this known matrix to the transformation contained in the DICOM registration object generated by the registration software, the matrix in eq. (4) has to be inverted, which can be done for example by using MINVERSE array function in Excel. Numerically,

(5) $$M^{-1}=\left[\begin{array}{cccc} 0.975 & 0.173 & 0.139 & -5.07\\ -0.161 & 0.983 & -0.087 & 17.29\\ -0.152 & 0.061 & 0.987 & -18.06\\ 0 & 0 & 0 & 1 \end{array}\right]$$

The translation vector ***T*** from the last column (transcribed to Table 2) can now be compared to the registration software‐generated one to obtain the registration errors along the cardinal axes. Note that the errors thus determined are only correct for the point at the coordinate system origin. The errors would vary at different points in the phantom depending on the angular misalignment. Since the reference frame origin of the Basic Anatomical Phantom is close to the geometrical center, we limited our reporting to that point. To determine the registration error at an arbitrary point, the full nominal transformation matrix would have to be applied first to its coordinates to determine the expected translations.

Due to the degenerate nature of 3D rotational transformation, angular error cannot be decomposed back to components corresponding to the individual axes. Nevertheless, the overall angular misalignment can be estimated using eigenvectors. The eigenvector of a rotational matrix determines the direction of the axis around which the composite rotation takes place (e.g., the line unchanged by the rotation). There is one real eigenvector for a 3 × 3 matrix. For the matrix in eq. (5), its coordinates are (−0.3165, −0.6251, 0.7135). Eigenvectors for a rotational matrix reported by the registration software can be calculated by using a function in one of the ubiquitous math software packages (Matlab, Mathematica) or a free online calculator (e.g., http://comnuan.com/cmnn01002/). The cosine of the angle between the expected and achieved rotational axes is immediately found from a dot product of the nominal unit eigenvector above and the one from the rotational matrix exported by the software. The angle between the eigenvectors quantifies overall misalignment between the known and calculated axes of rotation.

#### 2.B.4 Deformable registration

The quantitative deformable registration tests are enumerated in Table 3. The Report provides two dataset pairs for evaluation of DIR, using, in theory, two different methods of providing the ground truth transformation. The first is Basic Deformation Dataset 1. It is constructed from Basic Anatomical Dataset 1 by adding noise, translations, rotations, and deformation in the central region. It is stated in the Report that “evaluating the accuracy of the deformation phantom should be performed using the DICOM deformation vector field (DVF) files”.^1^ Unfortunately, this recommendation was not followed, and the ground truth DVF file provided in the Report's supplemental materials is in a proprietary binary format, making it unusable without the corresponding commercial software package.

**Table 3:** Deformable registration TRE tests

Case	Stationary dataset	Moving dataset	Error quantification method
15	Basic Anatomical Dataset 1 (CT)	Basic Deformation Dataset 1 (CT)	Contour comparison
16	Clinical 4DCT Dataset (phase 00)	Clinical 4DCT Dataset (phase 50)	Virtual fiducials‐TRE; Contour comparison
17	POPI Dataset 2 (phase 00)	POPI Dataset 2 (phase 50)	Virtual fiducials‐TRE; Contour comparison
18	POPI Dataset 6 (phase 00)	POPI Dataset 6 (phase 50)	Virtual fiducials‐TRE; Contour comparison
19–22	POPI Datasets 1,3–5 (phase 00)	POPI Datasets 1,3–5 (phase 00)	Virtual fiducials‐TRE
23–25	Clinical Abdomen cases (phase 00)	Clinical Abdomen cases (phase 50)	Contour comparison
26–28	Clinical Head and Neck cases (treatment planning CT)	Clinical Head and Neck cases (diagnostic CT)	Contour comparison

Constructing the ground truth DVFs is a nontrivial endeavor.^10^, ^11^ As a result, an alternative practical approach to Case 15 had to be developed. As an easy first step, the center of each of the three visible fiducials was identified on the target and deformed images, and the differences recorded as target registration errors (TRE). For a more comprehensive analysis, we segmented the datasets and compared the structures resulting from deforming the moving dataset to those manually drawn on the target (noisy) dataset. The analysis was done with the StructSure tool (Standard Imaging Inc. Middleton, WI, USA) based on the work by Nelms et al.^12^ However, of the menu of metrics available in the software, we chose only the one that could be, albeit with some effort, extracted manually from any radiotherapy planning/registration system. The pertinent values are the volumes of the deformed and target structures and of their overlap. From that, the Dice similarity coefficient (DSC)^13^ can be calculated as

(6) $$SC=\frac{2(V_{A}\cap V_{B})}{V_{A}+V_{B}}$$

where *V*_*A*_ and *V*_*B*_ are the volumes of the deformed and target structures and $V_{A}\cap V_{B}$ is their overlapping volume. On the other hand, determination of the mean distance between contour surfaces, which is another structure‐based metric recommended in the Report, is too time consuming for manual calculations and would require a specialized software tool. Fortunately, a formal statistical analysis in a recent publication^11^ suggests that DSC and structure volume are a strong predictor of the distance to conformity between contours, and the latter may be omitted as redundant.

The second DIR case provided in the Report, Clinical 4DCT Dataset (Case 16 in Table 3), is intended to be used with a TRE‐type quantification scheme. It has 300 virtual fiducials semiautomatically placed at bifurcation points identified on both end‐inhalation and end‐exhalation respiratory phases.^14^ The sets of Euclidian distances between the corresponding points on the deformed dataset and the target were analyzed as suggested in the Report. The DVF exported by the registration software was extracted from the DICOM object and applied to the fiducials’ coordinates on the first dataset to determine their position on the second one. Those positions were compared to the known fiducials’ coordinates on the second dataset. To facilitate this process, a C++ routine was developed, which can be obtained from the authors upon request. It was validated by manually identifying the corresponding coordinates of 10 randomly selected fiducial points and comparing the error to the program. The average difference in 3D displacement between the manual calculation and the C++ program was 0.15 ± 0.52 mm (1SD), with the range from −0.6 to 1.3 mm. This is adequate as the DVF voxel size reported by Mirada for this dataset is 1.94 × 1.94 × 5 mm^3^. Given the paucity of the deformable registration datasets provided in the report, six more Thoracic CT scan pairs of inhale/exhale respiratory phases, each with 100 manually placed virtual fiducials^15^, ^16^ were downloaded and analyzed in the same manner (Cases 17–22).

In addition, datasets from Cases 16–18 were segmented by a local expert (JC) on both respiratory phases and the deformed contours from the moving dataset compared to those drawn on the target, as described before. This allows for useful cross‐checking of the results between two independent approaches to geometrical registration error determination. This method of producing the contour pairs is not as refined as the ones described by Loi et al.^11^ but has the advantages of not requiring specialized software and perhaps being somewhat more realistic.

The Report recommends 10 clinical cases to be examined, without specifying a method of obtaining the ground truth. We felt that the seven thoracic cases described above were sufficient for that anatomical region. Therefore, we added three randomly selected abdominal (two extreme respiratory phases) and three head and neck (treatment planning vs. diagnostic) CT dataset pairs as examples. Contour comparison was again selected as a practical method of quantifying the TRE. The normal structures were segmented on each dataset by an expert, and the contour comparison routine described above was applied.

Finally, to assess the consistency of the deformable registration with respect to direction, the segmented datasets (Cases 16–18 and 23–28) were registered in the opposite direction and the DSC metrics were compared between the direct and reverse registrations.

## 3 RESULTS

### 3.A Registration errors — rigid translations only (Cases 1–9)

The ½ voxel dimensions for the Basic Phantom Dataset (Cases 1–5) were 0.35 × 0.35 × 1.5 mm^3^. The TRE along the cardinal axes for these translational tests exceeded those values in x‐ and y‐directions for the CT to PET (~1.3 mm) and both CT to MRI (~ 0.5 mm) registrations. The x‐ and y‐directions TREs for CT to CT and CT to CBCT registrations never exceeded 0.13 mm, and so did the z‐direction TRE for all modalities.

For the Basic Anatomical Dataset (translational Cases 6–9), the ½ voxel dimensions were 0.46 × 0.46 × 1.5 mm^3^, with the exception of the MRI, where the ½ transverse pixel size was 0.91 × 0.91 mm^2^. Only the CT to CT registration had all TREs below ½ of the corresponding voxel size. For the PET‐CT test, x‐ and y‐direction errors exceeded 1 mm, while for both MRI to CT registrations only the x error was above 1 mm.

### 3.B Registration errors — rigid translations and rotations (Cases 10‐14)

The dataset voxel dimensions for rigid translation/rotation cases followed the same pattern as for Cases 6–9, with the MRI transverse pixel size being twice as large as for all other modalities. Only the PET‐CT registration had the errors at the origin exceeding ½ voxel size, 1.2 and 1.3 mm for x‐ and y‐directions, respectively. The composite angular misalignment of the rotational axis ranged from 0.1° for CT to CT registration to 0.96° for PET‐CT, with the other combinations falling in between.

### 3.C Registration errors — deformable

#### 3.C.1 Basic Deformable Dataset 1 (Case 15)

**Figure 2:**
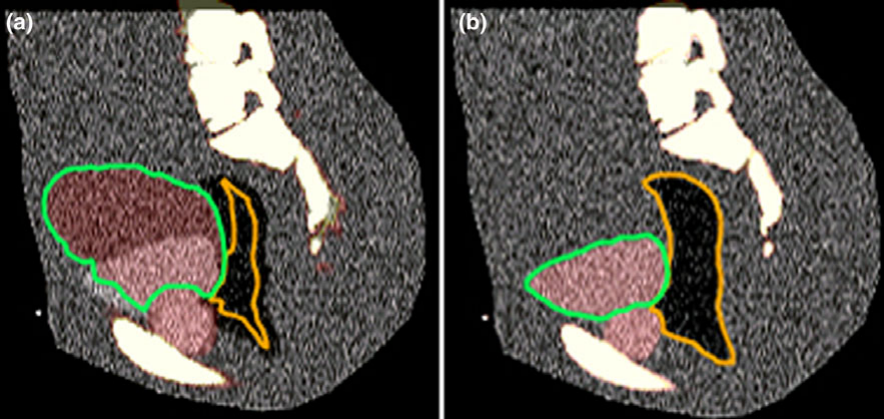
Deformable registration results for a noisy CT dataset (Case 15) with the Optical Flow (a) and Mutual Information (b) algorithms.

The optical flow‐based “CT Deformable” Mirada algorithm produced grossly erroneous results for Case 15 [Fig. 2(a)]. The structures such as bladder and rectum are substantially distorted. Voxel intensity dissimilarities caused by artificially added noise make this intensity‐based algorithm inadequate for the task. Subsequent testing for Case 15 was done with the CT to CT part of the Mutual Information‐based “Multimodal Deformable” algorithm, which produced visually acceptable results [Fig. 2(b)].

The 3D TRE errors between the target and deformed images were 1.1, 3.0, and 1.2 mm for the bladder, rectum, and prostate fiducials, respectively. The relatively high rectum fiducial TRE comes predominantly from the 3 mm misalignment in the z (superior‐inferior) direction.

The results of the contour similarity analysis for Case 15 are detailed in Table 4.

**Table 4:** Comparisons between the pertinent contours deformed from the moving dataset and those drawn on the target

ROI	DSC	Volume deformed (cc)	Volume target (cc)	Common Volume (cc)
Prostate	0.929	34.2	33.2	31.3
Bladder	0.957	239.2	224.5	221.8
Rectum	0.949	182.6	166.1	165.4
Femur_L	0.977	288.3	281.8	278.5
Femur_R	0.981	285.4	278.3	276.4
SV_LT	0.878	3.4	3.5	3.03
SV_RT	0.811	3.6	4.1	3.11

DSC, Dice Similarity Coefficient.

Also shown are total volumes for each subset of contours and the breakdown of volumetric differences, to demonstrate how DSC is calculated.

All contours, except for the “seminal vesicles”, show DSC well above the level considered acceptable in the Report (0.8–0.9).^1^ The “seminal vesicles” are small, low‐contrast structures and their low DSCs are mostly due to the inability of the observer to properly identify them on the noisy target image. With the very high Dice coefficients and maximum differences between the contours being of the order of one voxel size, this test was considered successful.

#### 3.C.2 Clinical Thoracic deformable registration (Cases 16‐22)

For the cases in this section, the Optical Flow “CT Deformable” Mirada algorithm was used. It is the primary algorithm intended for CT to CT registration and also provides better spatial resolution of the DICOM‐exported DVF. It is apparent from Table 5 that Mirada does not meet the Report recommendations of the mean TRE <2 mm and maximum <5 mm. At the same time, the contours for the major organs on a sample of segmented thoracic cases (16–18) overlap quite well (Table 6).

**Table 5:** Target registration error statistics for Thoracic cases 16–22

Case	Mean TRE±1SD (mm), Mirada	Max TRE (mm)
16	6.5 ± 8.1	29.0
17	4.5 ± 2.3	11.6
18	8.9 ± 3.5	21.3
19	5.6 ± 3.8	23.2
20	5.5 ± 4.3	27.3
21	4.1 ± 2.4	15.4
22	3.4 ± 1.7	10.1

**Table 6:** Thoracic Dice Similarity Coefficients (DSC) between the individual organ contours drawn on a respiratory phase (0% and 50%) and those propagated form the deformably registered different phase. Results are presented for both registration directions

Case	16		17		18		Ave	1SD
ROI	DSC 0→50	DSC 50→0	DSC 0→50	DSC 50→0	DSC 0→50	DSC 50→0		
Aorta	0.91	0.93	0.92	0.92	0.93	0.93	0.92	0.01
Esophagus	0.81	0.82	0.80	0.79	0.85	0.85	0.82	0.03
Heart	0.93	0.94	0.92	0.93	0.95	0.95	0.94	0.01
Lung_L	0.99	0.99	0.96	0.97	0.98	0.98	0.98	0.01
Lung_R	0.99	0.98	0.98	0.98	0.99	0.99	0.99	0.01
Spleen	0.92	0.94	0.93	0.95	0.96	0.96	0.94	0.02
Sternum	0.90	0.90	0.89	0.89	0.91	0.91	0.90	0.01
Stomach	0.87	0.90	0.94	0.93	0.79	0.79	0.87	0.07
Trachea	0.87	0.89	0.91	0.93	0.93	0.93	0.91	0.03

#### 3.C.3 Clinical Abdominal cases (Cases 23–25)

The difference in the abdominal datasets, as in the thoracic ones above, is that they belong to the two extreme respiratory phases. The DSC results for the major abdominal contours are presented in Table 7. For all organs except the pancreas, the overlap can be characterized as excellent (DSC ≥ 0.90). The average pancreatic DSC is fair at 0.79, with two registrations falling below 0.75. The pancreas is smaller than the other organs in the table.

**Table 7:** Abdominal DSCs between the directly drawn and deformably propagated major organ contours on two respiratory phases (0 and 50%). The results for both registration directions are presented

Case	23		24		50		Ave	1SD
ROI	DSC 0→50	DSC 50→0	DSC 0→50	DSC 50→0	DSC 0→50	DSC 50→0		
Heart	0.95	0.95	0.95	0.95	0.95	0.95	0.95	0.00
Kidney_L	0.94	0.94	0.90	0.90	0.92	0.92	0.92	0.02
Kidney_R	0.94	0.94	0.94	0.94	0.93	0.93	0.94	0.01
Liver	0.97	0.97	0.94	0.95	0.96	0.96	0.96	0.01
Pancreas	0.88	0.88	0.73	0.74	0.76	0.77	0.79	0.07
Spleen	0.93	0.93	0.90	0.90	0.90	0.90	0.91	0.02
Stomach	0.95	0.95	0.92	0.92	0.90	0.92	0.93	0.02

#### 3.C.4 Clinical Head and Neck cases (Cases 26–28)

The main challenge in aligning the diagnostic and treatment planning HN image sets is the flexion of the neck, which requires substantial deformation. Additionally, the diagnostic datasets include contrast media, particularly evident in major blood vessels. However, the vessel and major muscle alignment was visually checked and deemed very close. The results of the DSC between the drawn and warped contours in both directions are presented in Table 8. With the exception of inferior, mid, and superior pharyngeal constrictors (IPC, MPC, SPC), the average level of overlap per organ (DSC ≥ 0.75) can be considered acceptable as quoted by Loi et al.,^11^ although below the recommendations of the Report (0.8–0.9).^1^ The pharyngeal constrictors are small, thin, low‐contrast structures adjacent to air cavities, all of which makes it challenging for the software to align them properly. The average DSC for those structures varies from 0.39 to 0.69, and in one case (26) the original and deformed MPCs (Case 26) nearly do not overlap at all.

**Table 8:** Head and Neck DSCs between the diagnostic (D) and treatment planning (RT) CT scans for a sample set of commonly segmented normal structures

Case	26		27		28		Ave	1SD
ROI	DSC RT→D	DSC D→RT	DSC RT→D	DSC D→RT	DSC RT→D	DSC D→RT		
BrainStem	0.79	0.73	0.75	0.64	0.78	0.82	0.75	0.06
Cerebellum	0.88	0.63	0.90	0.82	0.65	0.89	0.80	0.12
IPC	0.67	0.62	0.64	0.67	0.77	0.76	0.69	0.06
Larynx	0.81	0.78	0.68	0.78	0.82	0.84	0.79	0.06
Mandible	0.82	0.81	0.90	0.88	0.91	0.95	0.88	0.05
MPC	0.03	0.08	0.49	0.46	0.62	0.64	0.39	0.27
OralCavity	0.76	0.76	0.83	0.85	0.86	0.90	0.83	0.06
Parotid_L	0.76	0.73	0.79	0.83	0.83	0.84	0.80	0.04
Parotid_R	0.78	0.75	0.84	0.86	0.83	0.84	0.82	0.04
SPC	0.44	0.44	0.59	0.60	0.78	0.51	0.56	0.13
SpinalCord	0.80	0.80	0.78	0.78	0.65	0.82	0.77	0.06

### 3.D Consistency with respect to registration direction

The robustness of deformation with respect to direction depends on the criteria and follows the quality of the corresponding registration metrics. For Thoracic case 16, for example, the misalignment of the virtual fiducials is rather large (Table 5). Similarly, the mean (Δx, Δy, Δz) are unstable with direction of registration and change from (−1.6, −2.3, 18.6 mm) for the 0% to 50% deformation to (−0.02, 1.2, −5.1 mm) for the opposite one. On the other hand, the DSCs between the thoracic and abdominal contours in Tables 6 and 7 are rather high and do not change meaningfully with direction. The HN DSCs show more random variation, as the contour overlap is generally lower (Table 8).

## 4 DISCUSSION

In stark contrast, for example, with the dose calculation algorithms,^17^ the guidance literature on validation of image registration software, particularly DIR, is still in its infancy. The issue is rather complex, as the apparent registration success or failure depends on multiple variables, such as the algorithm, site, metrics, and clinical goals. The Report provides a reasonable suite of virtual phantoms and criteria for rigid registration validation. In this paper, we elaborated on their detailed application to a particular commercial software package. Even in these simplest cases, the strict criterion of ½ voxel size registration accuracy is not met in every case, although the overall error magnitude is reasonably small (≤1.3 mm in any single direction). In general, evaluation of the rigid registration is straightforward because the expected result is unambiguous and easily quantified without specialized software tools.

On the other hand, for deformable registration the Report suffers from the same problem as the field in general — the scarcity of well‐characterized ground truth information. In addition to the digital phantoms, the Report recommends “evaluation of the registration accuracy … using example clinical datasets”,^1^ while providing little specific guidance. A survey of the literature on validation of commercial DIR algorithms reveals a number of conceptual approaches to the problem. In decreasing order of generality, they are: (a) comparison of the deformation vector field (DVF) with the ground truth one^10^, ^18^; (b) examining propagation of the large number of anatomical landmarks (points) to determine TRE^14^, ^15^, ^19^; (c) investigating the overlap of the deformably propagated contours with the known segmentation results^11^, ^20^, ^21^, ^22^; and, finally, (d) physical phantom evaluations.^23^, ^24^, ^25^ It appears that comparison of the deformation vector fields should be the most comprehensive method of validating DIR. In reality, generating clinically meaningful ground truth DVFs on clinical datasets (as opposed to phantoms) is not easy and requires specialized software tools.^10^, ^26^ Frequently, the DIR software has to manipulate images to account for missing or extra voxels on one set compared to another. Therefore, establishing a one‐to‐one voxel correspondence, necessary for a true standard DVF, is often challenging. Typically, the datasets have to be artificially generated,^26^ but that could lead to questions of their real‐world validity. In practice, public domain ground truth datasets pairs with DVFs are few and far between. In a single digital phantom case provided in the Report, such DVF is in a proprietary binary format not readable without the specific commercial software package. As a result, no DVF comparisons were performed in this work, which is also true for the majority of published papers on commercial DIR software evaluation.

Analyzing the TRE for a large number (hundreds) of virtual fiducials is the step‐down from the DVF analysis for every voxel, but it is still capable of producing a fairly detailed picture of the registration accuracy within an organ (typically the lungs^14^, ^15^, ^16^). One digital dataset pair with the corresponding sets of fiducials from Ref. [14] is provided in a supplement to the Report. We additionally analyzed six publicly available, conceptually similar datasets.^15^, ^16^ The Mirada DIR algorithm performed poorly on these tests, demonstrating the mean and maximum TRE values in each case far in excess of 2 and 5 mm criteria, respectively, suggested in the Report.

The next step in simplification of the DIR quality analysis is contour comparison. Now, the randomly selected fiducial points are replaced by the organ(s) surface contours, and the point‐to‐point TRE values are replaced by the less‐specific contour overlap or closeness metrics. On the positive side, this type of test can be easily designed by virtually any facility, as all that is required is a pair of expertly segmented clinical datasets. Our DIR software performed well in these tests for major thoracic and abdominal organs segmented on two respiratory phases, and fairly for the HN cases with differences in neck flexure. This underscores that the requirements for faithful contour propagation are not synonymous to, and may in fact be disparate from, the requirements for volumetric spatial accuracy of image registration.^24^ Hybrid DIR models are being proposed to address this issue.^27^ In our case, the DIR algorithm appears to be adequate for contour propagation but is questionable at best for applications requiring the fidelity of the volumetric DVF, such as deformable dose accumulation.^24^, ^27^, ^28^, ^29^ Finally, it is fair to say that DIR evaluations with physical phantoms are not practically feasible in the majority of the radiotherapy clinics.

## 5 CONCLUSIONS

Given the wide availability of commercial image registration software, the AAPM TG‐132 Report^1^ is a useful, albeit far from complete, step toward providing a medical physicist with the knowledge, tools, and criteria for validating those algorithms in the clinic. We demonstrated how a number of suggested quantitative tests can be performed using only publicly available tools. However, for deformable registration, the Report on the practical level provides more questions than answers. There is a great need for a universally available, comprehensive library of digital datasets with the ground truth deformation data. A good example of a related recent project relying on public domain software and providing downloadable datasets would be the work by Nyholm et al.^30^ Furthermore, it may not be realistic to expect a clinical physicist to perform validation of a DIR package for a full variety of clinical sites and use scenarios. A more practical approach may be for the software vendors to provide a comprehensive, objective set of characterization and validation data for their algorithms, from which at least an initial approximation of fitness for a particular task could be inferred.

## CONFLICT OF INTEREST

The authors have no relevant conflict of interest to report.
